# Editorial: Deepening the relationship between diabetes, oral health and periodontal disease

**DOI:** 10.3389/froh.2025.1752592

**Published:** 2026-01-09

**Authors:** Razia Abdool Gafaar Khammissa, Oelisoa Mireille Andriankaja

**Affiliations:** 1 Department of Periodontics and Oral Medicine, University of Pretoria Oral and Dental Hospital, Pretoria, South Africa; 2 Center for Oral Health Research (COHR), University of Kentucky College of Dentistry, Lexington, KY, United States

**Keywords:** diabetes mellitus, periodontal disease, microbial dysbiosis, immuno-inflammatory response, reactive oxygen species

**Figure 1:**
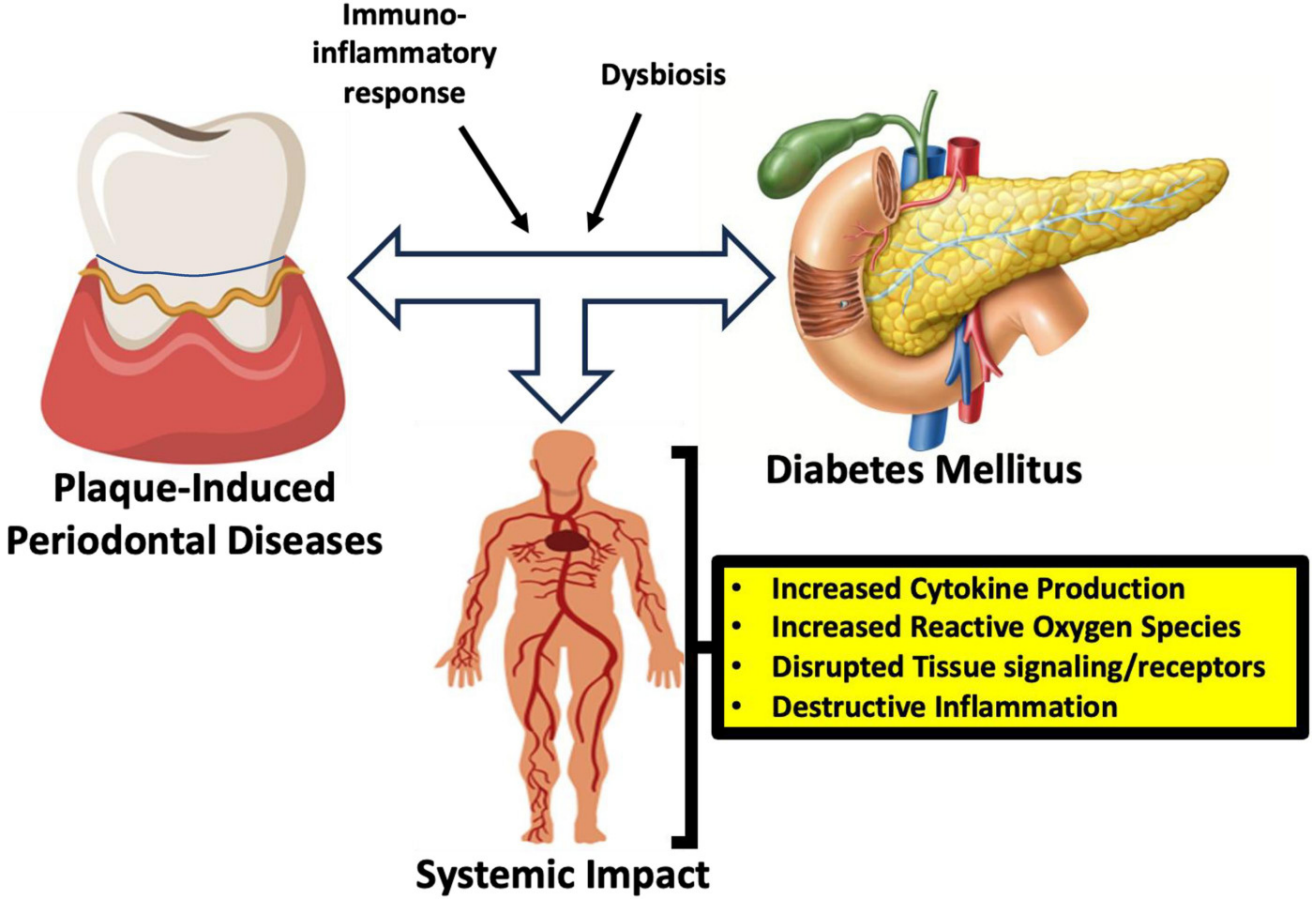
Conceptual representation of the bidirectional relationship between diabetes and periodontal disease, illustrating key mediating mechanisms including microbial dysbiosis, systemic inflammation, and metabolic dysfunction.

The complex interplay between diabetes mellitus and periodontal disease has been recognized for decades, yet emerging research continues to uncover additional biological and behavioral mechanisms linking these two chronic conditions (Figure 1). This special issue of Frontiers in Oral Health, *Deepening the Relationship between Diabetes, Oral Health and Periodontal Disease*, brings together nine contributions that collectively expand our understanding of how metabolic dysregulation and oral inflammatory disease intersect across pediatric and adult populations.

A central theme across these studies is the set of shared pathophysiological pathways— rooted in inflammation, microbial dysbiosis, and immune modulation—that underlie both diabetes and periodontal disease. The article “*Periodontal Health and Metabolic Status of Type 1 Diabetic Children and Adolescents*” shows that even in young patients with type 1 diabetes, suboptimal glycemic control is associated with worsened periodontal parameters. Similarly, “*Cross-sectional association among dietary habits, periodontitis, and uncontrolled diabetes in Hispanics: the LLIPDS study*” highlights the influence of dietary patterns and metabolic control on periodontal outcomes.

Microbiological and molecular perspectives further enrich this picture. “*Microbiomic insights into the oral microbiome's role in type 2 diabetes mellitus*” outlines methodological standards for future oral microbiome research, while “*The impact of Filifactor alocis on the severity of periodontitis among diabetic and non-diabetic patients*” identifies this emerging pathogen as a potential mechanistic link between hyperglycemia, immune dysregulation, and periodontal tissue destruction.

Systemic interactions are also a key focus. The study “*Longitudinal study on metabolic abnormalities and diabetes risk in normal-weight Japanese adults*” emphasizes that metabolic dysfunction and diabetes risk may manifest independently of obesity. Cardiovascular comorbidities are explored in “*Coronary atherosclerosis and periodontitis have similarities in their clinical presentation*” and “*Association of periodontitis and tooth loss with extent of coronary atherosclerosis in patients with type 2 diabetes mellitus*” both of which highlight converging inflammatory mechanisms across vascular and periodontal tissues.

Population-level analyses add broader context. “*Oral health's role in diabetes risk: a crosssectional study with sociodemographic and lifestyle insights*” identifies behavioral, demographic, and socioeconomic contributors to both diabetes and oral health outcomes. Meanwhile, “*Rural–urban disparities in the incidence and treatment intensity of periodontal disease among patients with diabetes*” highlights persistent inequities in access to oral healthcare and disease management.

Collectively, these contributions demonstrate the multifaceted nature of the relationship between diabetes and periodontitis, spanning biological, behavioral, and systemic dimensions. At the same time, the predominance of cross-sectional designs within this special issue limits conclusions about directionality and causality. Although the evidence consistently suggests a bidirectional association, the extent to which periodontal disease contributes to the development or progression of diabetes—and vice versa—remains to be clarified through longitudinal and mechanistic research.

Future studies should therefore incorporate long-term designs, multi-omics approaches, and interdisciplinary care models to elucidate causal pathways, identify predictive biomarkers, and determine whether integrated medical–dental interventions can improve both glycemic and periodontal outcomes. These limitations are consistent with broader trends in the literature. Although the bidirectional relationship between periodontal disease and diabetes mellitus has been widely acknowledged by Preshaw et al. (2012), much of the existing evidence base is constrained by cross-sectional designs, heterogeneity in diagnostic criteria, and insufficient control of confounders. Consensus reports have likewise emphasized the need for methodological standardization and more rigorous study designs to clarify causal pathways as suggested by Sanz et al., 2018. Microbiome-focused studies, such as Casarin et al. (2013), further highlight the potential role of microbial dysbiosis in linking hyperglycemia to periodontal inflammation, yet they also demonstrate the limitations of small sample sizes and the absence of longitudinal follow-up. Collectively, these gaps reinforce the necessity for long-term cohort studies, multi-omics approaches, and well-designed clinical trials to determine whether integrated medical– dental interventions can meaningfully modify both glycemic control and periodontal outcomes.

